# Metabolic biomarker in oral squamous cell carcinoma – a comprehensive review

**DOI:** 10.3205/iprs000190

**Published:** 2025-04-16

**Authors:** Karsten Schreder, Claudia Wickenhauser, Matthias Kappler, Frank Tavassol, Alexander W. Eckert

**Affiliations:** 1 Martin Luther University Halle-Wittenberg, University Hospital, Department of Oral and Maxillofacial Plastic Surgery, Halle (Saale), Germany; 2 Dental Office, Maxillofacial Surgery, Alexander Raue, Dres. Schäfer, Halle (Saale), Germany; 3 Martin Luther University Halle-Wittenberg, University Hospital, Institute of Pathology, Halle (Saale), Germany; 4 Paracelsus Medical University Nuremberg, Department of Oral and Maxillofacial Plastic Surgery, Nuremberg, Germany

**Keywords:** metabolic biomarker, OSCC, prognosis, survival

## Abstract

Oral squamous cell carcinoma (OSCC) is one of the most common malignant tumors worldwide with an increasing incidence. The surgical treatment is challenging and often requires the entire repertoire of plastic surgery. Diagnostically only a few crucial parameters are in use and even less for an individual and specific drug targeting. An individualised prognostic calculation is unavoidable to be able to adapt very complex surgical processes to an acceptable level. Unfortunately, the classic TNM system and grading are no longer sufficient, especially for individualized prognosis. Moreover, despite advances in treatment, studies have shown that the prognosis of patients with OSCC in terms of survival rate has not improved significantly, which is mainly due to the presence of treatment-resistant OSCC. Therefore, the identification of new, reliable biomarkers for early diagnosis and drug targets of OSCC is urgently needed. Meanwhile, the abundance of potential biomarkers for OSCC is difficult to keep track of. Therefore, the aim of the article was to provide an overview of articles listed in Pubmed^®^ that deal with the topic of biomarkers in oral squamous cell carcinoma, focusing in particular on the topic metabolism. Another question of this study was to set the focus on essential additive metabolic biomarkers, which can also be easily used in clinical routine.

## Abstract (de)

Das orale Plattenepithelkarzinom (OSCC) ist einer der häufigsten bösartigen Tumoren weltweit mit steigender Inzidenz. Die chirurgische Behandlung ist anspruchsvoll und erfordert oft das gesamte Repertoire der plastischen Chirurgie.

Diagnostisch sind nur wenige entscheidende Parameter relevant und noch weniger für eine individuelle und spezifische Medikamenten-Zielgruppenansprache. Um sehr komplexe chirurgische Prozesse auf ein akzeptables Maß anpassen zu können, ist eine individualisierte Prognoseberechnung unumgänglich.

Leider reichen das klassische TNM-System und die Einstufung insbesondere für eine individualisierte Prognose nicht mehr aus. Darüber hinaus haben Studien trotz Fortschritten in der Behandlung gezeigt, dass sich die Prognose von Patienten mit oralen Plattenepithelkarzinom im Hinblick auf die Überlebensrate nicht wesentlich verbessert hat, was hauptsächlich auf das Vorhandensein von behandlungsresistentem oralen Plattenepithelkarzinomen zurückzuführen ist.

Daher ist die Identifizierung neuer, zuverlässiger Biomarker für die Frühdiagnose und Wirkstoffziele von OSCC dringend erforderlich. Mittlerweile ist es schwierig, den Überblick über die Fülle an potenziellen Biomarkern für OSCC zu behalten.

Ziel des Artikels war es daher, einen Überblick über in Pubmed^®^ gelistete Artikel zu geben, die sich mit dem Thema Biomarker beim oralen Plattenepithelkarzinom beschäftigen und dabei insbesondere auf das Thema Stoffwechsel eingehen. Eine weitere Fragestellung dieser Studie bestand darin, den Fokus auf essenzielle additive metabolische Biomarker zu legen, die auch im klinischen Alltag problemlos eingesetzt werden können.

## 1 Introduction

**Figure 1:**
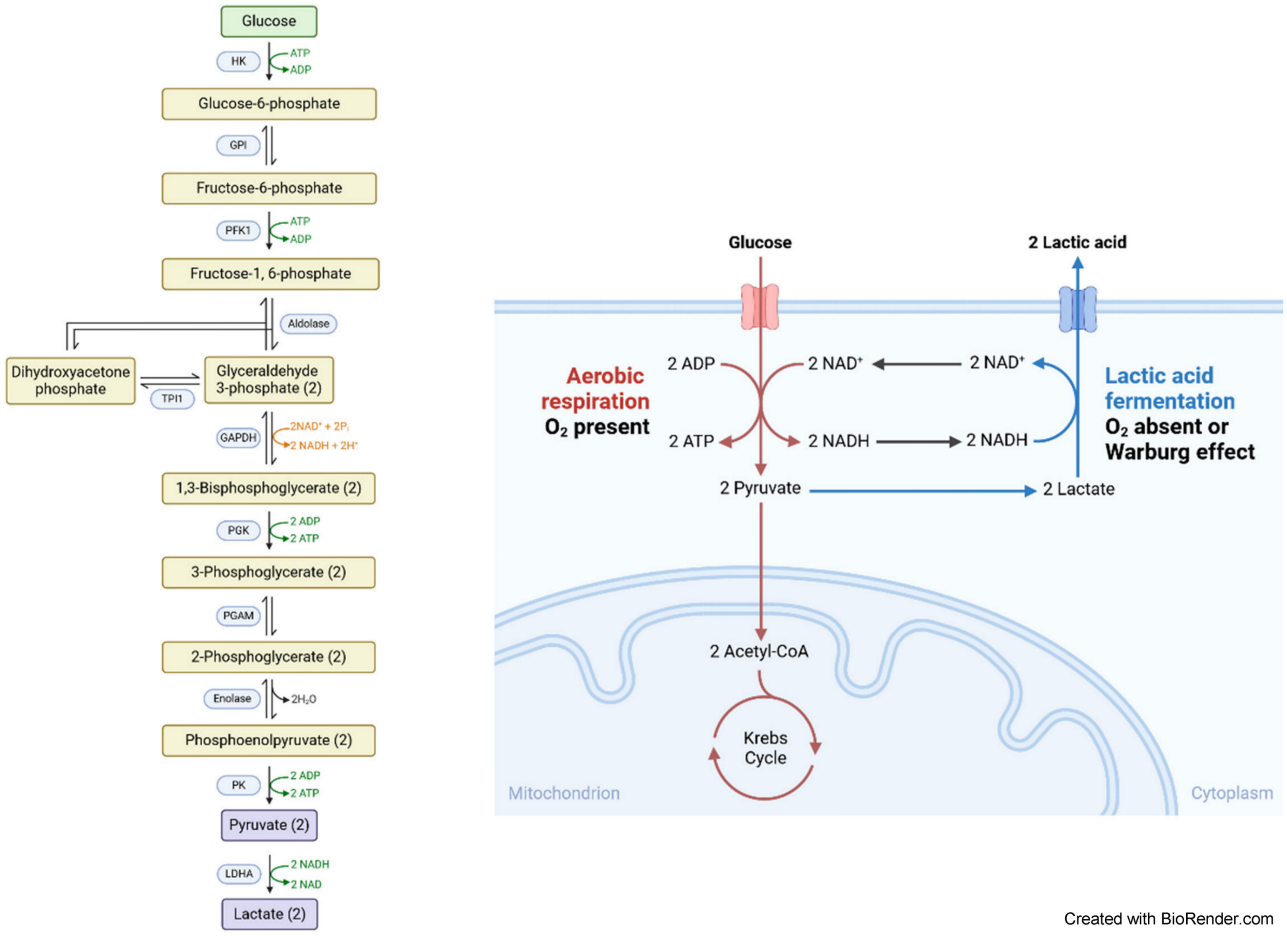
Glycolysis pathway Normally, the glucose metabolism ends in mitochondrial respiratory chain and TCA (citric acid cycle). In tumors, among them OSCC, it is limited at lactate! Abbreviations: GAPDH – glyceraldehyde-3-phosphate dehydrogenase; HK – hexokinase; LDHA – lactate-dehydrogenase A; PFK1 – phosphofructokinase; PGAM1 – phosphoglycerate mutase 1; PGK1 – phosphoglycerate kinase 1; TPI1 – triosephosphate-isomerase

Oral squamous cell carcinomas (OSCC) are among the twenty most frequent human malignancies worldwide (Global Cancer Observatory, https://gco.iarc.fr/ [accessed 2024 Jun 25]). Treatment is usually surgical with resection of the tumor and neck dissection. The resulting defects are often not insignificant and require complex reconstruction procedures. From a plastic surgery perspective, it would often be desirable to minimize the extent of surgical methods, taking into account various risk factors [1]. This brings biomarkers into focus in order to be able to calculate the individual prognosis in detail. According to GLOBOCAN estimates, there is an overall increase in the incidence and mortality of this type of cancer in Europe [2]. Moreover, the 5-year survival rate has stagnated at around 50% for over four decades. This indicates that TNM and grading alone are not sufficient for an individualized prognosis [3], [4]. Consequently, there is an urgent need to establish new, additional prognostic factors (“biomarker”) to characterize the individual aggressive potential of the OSCC in detail on the one hand, and on the other hand individualize the therapeutical strategy. In an earlier analysis, we were able to certify the excellent quality of head and neck tumor centers in Germany [5]. However, our results showed that biomarkers have not yet been used primarily in clinical routine in the majority of tumor centers. One of the first promising initial additive biomarkers is Epithelial Growth Factor Receptor (EGFR) [6]. However, on the one hand EGFR is not uniformly expressed on all OSCC tumor cells. Moreover, mutations in various domains of the EGFR gene not only alter drug binding dynamics giving rise to resistance have been described [7]. On the other hand and especially emphasized, the molecular pathogenesis of OSCC is very complex and results from a multitude of events that include the interplay between genetic mutations and altered transcript, protein and metabolite levels [8]. Intriguingly, of particular interest are biomarkers that are involved in tumor metabolism. This is due to the astonishing metabolic adaption of many tumors, which was first demonstrated by Otto Warburg and colleagues at the beginning of the 20^th^ century [9]. The Warburg effect characterizes an alteration of the glucose metabolism of tumor cells that leads to the production of large amounts of lactate. This requires a modulation of the expression of various biomarkers that influence this adaptive metabolism, which then become of course detectable. For a better understanding, the complexity of the glucose metabolism in the tumor cell should be emphasized more in detail. Therefore, an overview of glycolysis in tumors is presented in Figure 1 (Fig. 1).

The aim of the present study was to analyze and develop a comprehensive analytical review to determine additive prognostic factors in oral squamous cell carcinoma, especially for metabolic parameters.

## 2 Material and methods

We reviewed Pubmed^®^ within two and a half years from January 2022 to June 2024. All articles related to biomarkers in oral squamous cell carcinoma were checked fundamentally. “Oral squamous cell carcinoma, biomarkers and prognosis” (Boolean operators only AND), these were the keywords of our search.

**Figure 2:**
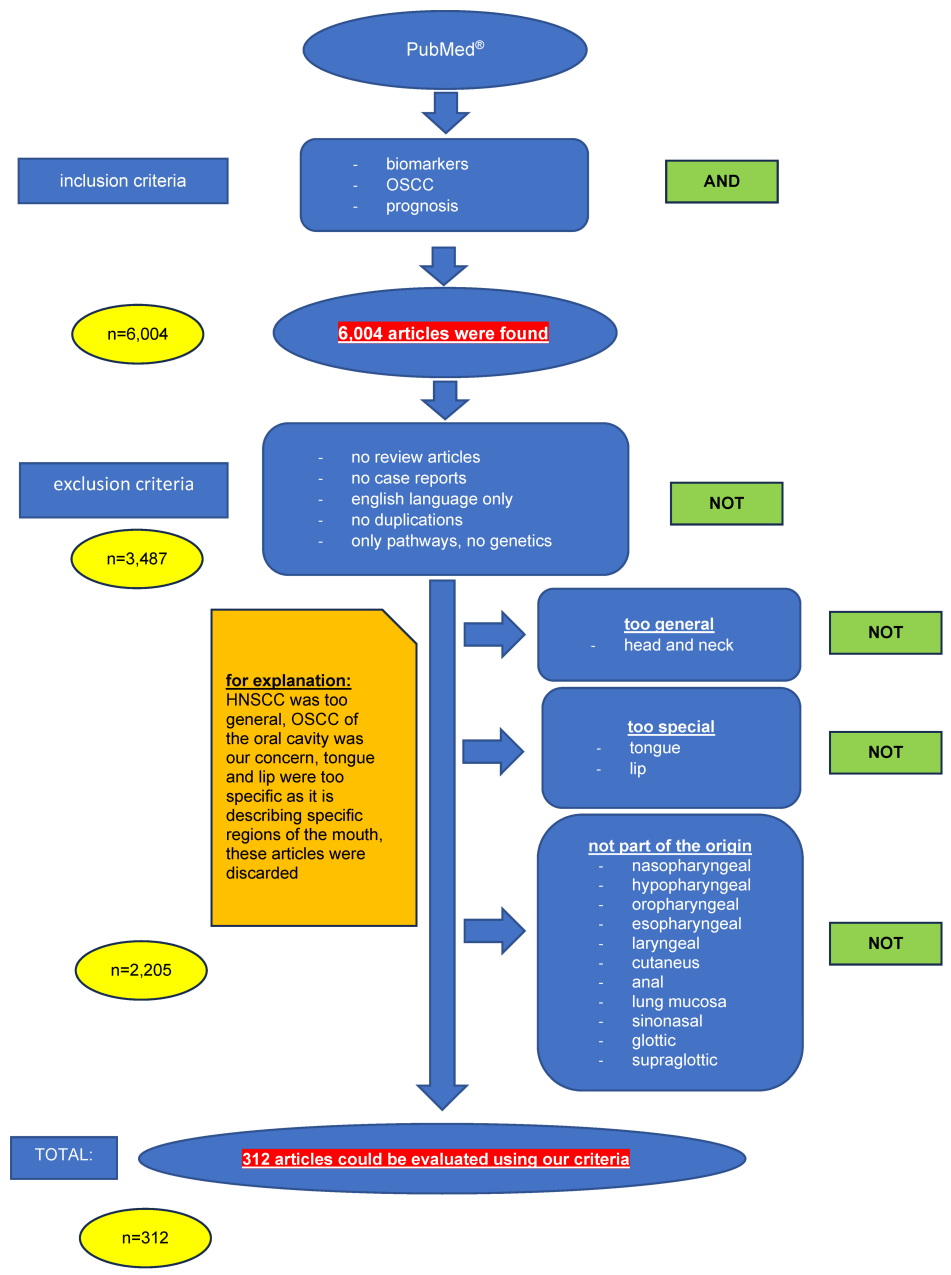
Graphic of inclusion and exclusion criteria in the PubMed^®^ search

We set the following exclusion criteria: no review articles, no case reports, no paper written in a language other than English, no duplications, only pathways and no genetics (Figure 2 (Fig. 2)). 

In addition, all papers that were review articles or case reports were filtered out. Moreover, the articles that existed twice (described the same biomarker, only one article on the specific biomarker was reported here and was included in our study) were also eliminated. Furthermore, we were only interested in the pathways and not the genetic route (Boolean operators only NOT). In addition, the articles that were not written in English were excluded. All articles were successively assigned to these criteria using the NIH^®^ (National Library of Medicine) algorithm. All inclusion and exclusion criteria are listed as flow diagram and shown graphically in Figure 2 (Fig. 2). 

## 3 Results

The Pubmed search with the keywords “oral squamous cell carcinoma AND biomarkers AND prognosis” resulted in 6,004 articles found. Applying the exclusion criteria no review articles, no case reports, all articles in English, no duplications, only pathways and no genetics 2.517 publications remained. However, applying all exclusion criteria (see Figure 2 (Fig. 2)) only 312 (5.2%) articles remained for further systematic analysis.

**Figure 3:**
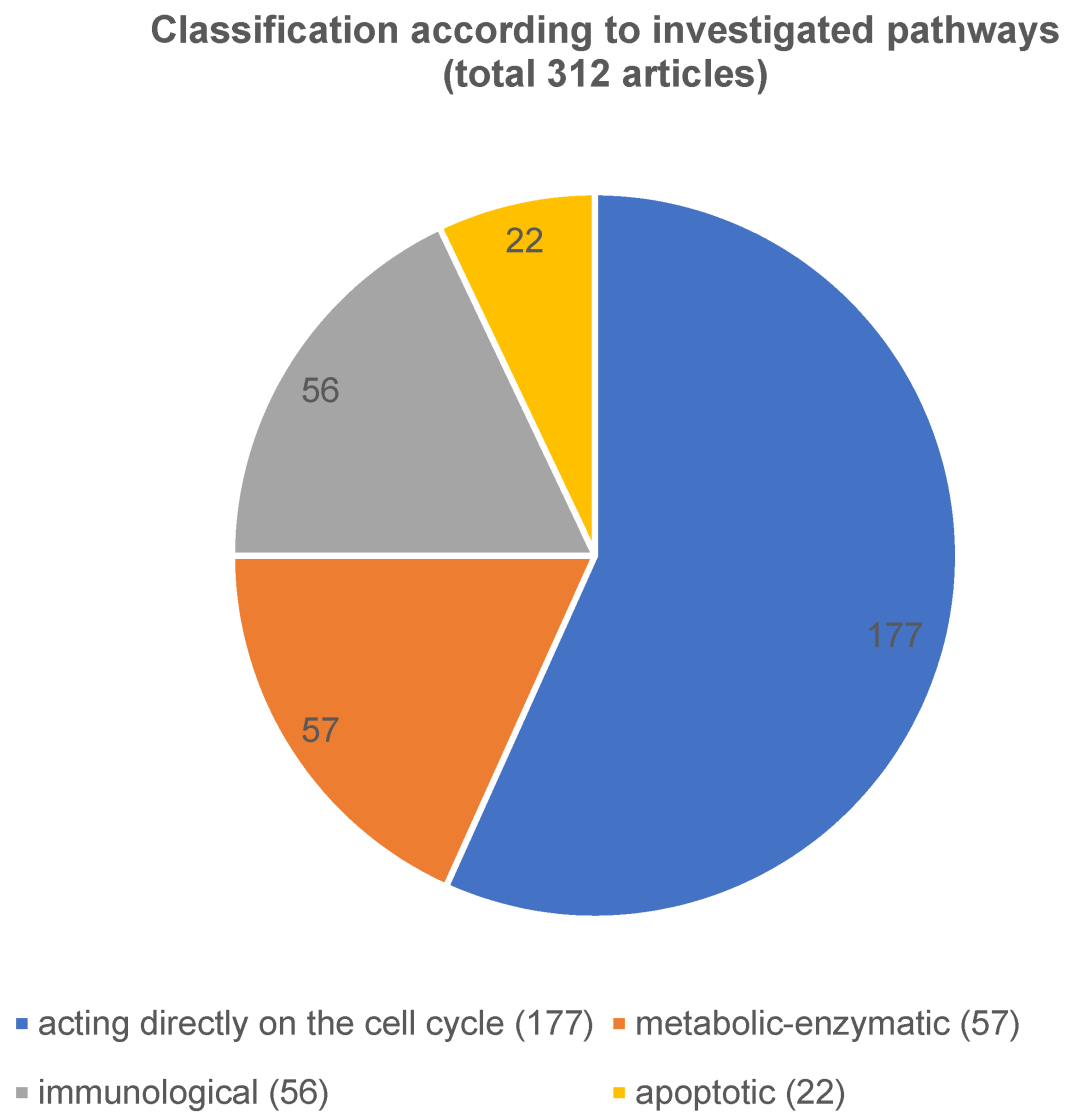
Classification according to investigated pathways

The publications were classified according to the different points as follows into 4 large groups: i) cell cycle, ii) metabolic, iii) immunological relevant proteins as well as iv) proteins involved in apoptosis. Figure 3 (Fig. 3) provides an overview of all 312 relevant proteins in accordance to their classification of the different pathways. 

**Figure 4:**
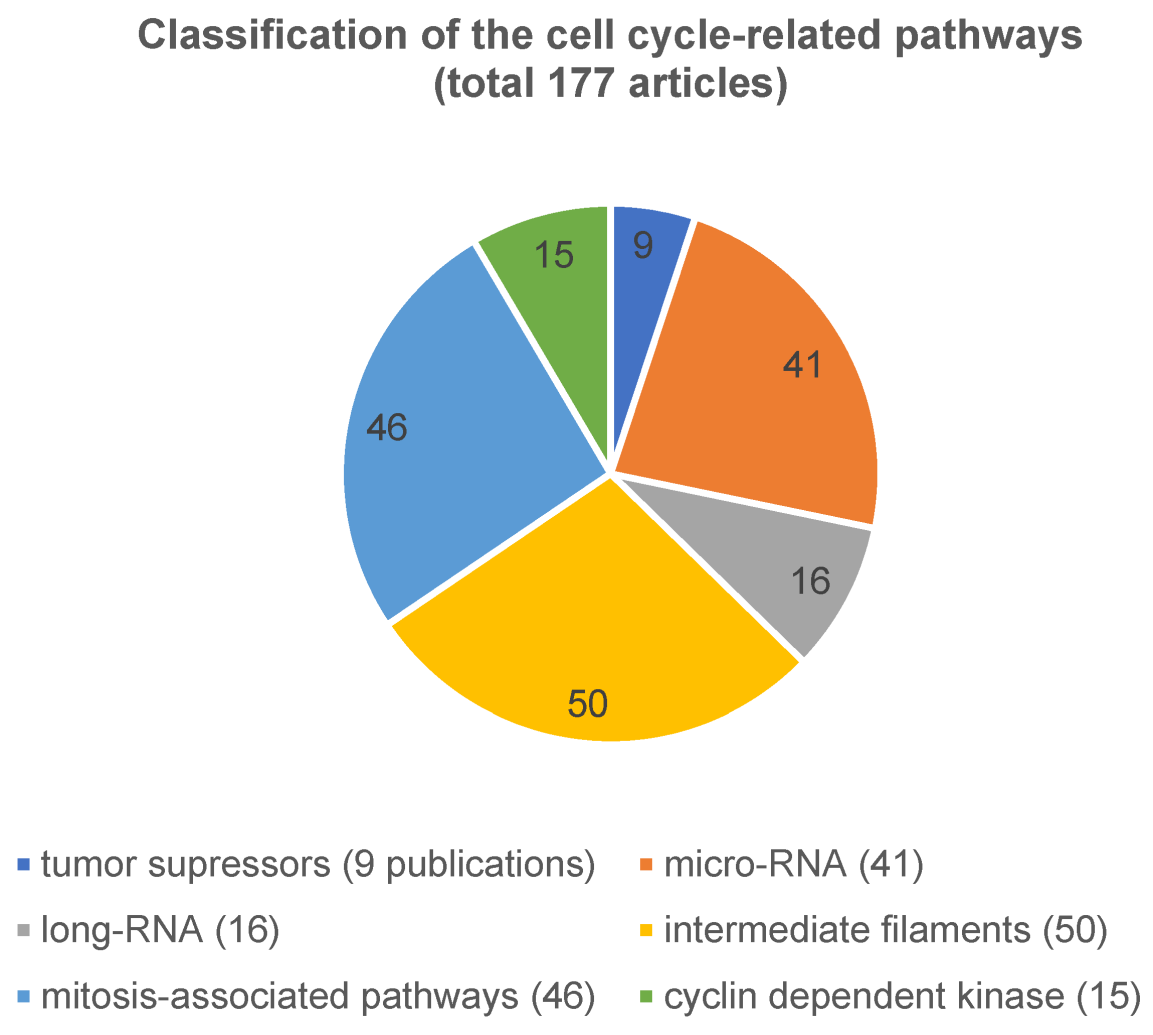
Classification of the cell cycle-related pathway

i) The first and largest group summarizes all aspects acting directly on the cell cycle, 177 articles were identified (Figure 4 (Fig. 4)). These were tumor suppressors (n=9), micro-RNA (n=41), long-RNA (n=16), and others (n=111). These ‘others’ mentioned include cell membrane structure, adhesion of the cells to each other like intermediate filaments, cyclin dependent kinase or mitosis-associated pathways. 

**Figure 5:**
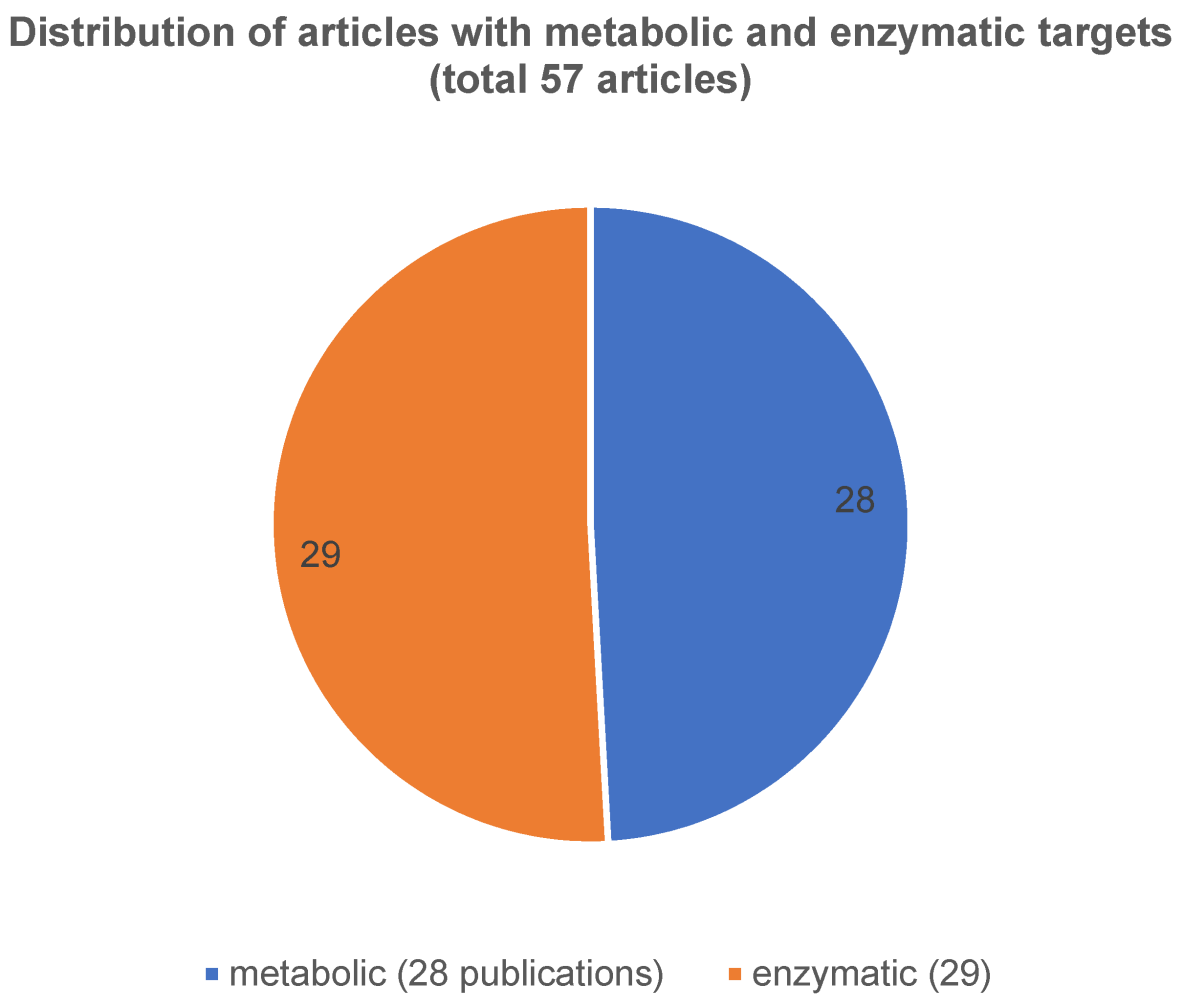
Articles with metabolic and enzymatic targets

The pathway that directly affects the cell cycle has been published most frequently (177 articles (57%)). Of these, 50 of the 177 articles described the structure of the cell membrane and the adhesion of cells, which corresponds to around 28%. Moreover, 41 of 177 articles (23%), had micro-RNA (for example: [10], [11], [12], [13], [14], [15], [16], [17] as topic. In addition, mitosis-associated pathways were related to only 46 of the 177 articles (26%). In comparison, tumor suppressor genes (9 publications of the 177, 5%), cyclin dependent kinase markers (15 publications of the 177, 8%) and lncRNA (16 publications of the 177, 9%) (publishing examples: [18], [19], [20], [21] were less studied.

ii) The second very interesting and quite homogeneous group (57 publications in total) can be subdivided into a metabolic-enzymatic pathway of which 28 articles were assigned to metabolism and 29 articles to enzymatic classified (Figure 5 (Fig. 5)). Comparing these investigated pathways, it is noticeable that the metabolic-enzymatic pathway is published in 57 (18%) of the 312 articles which corresponds to one fifth of the articles found, according to our criteria. As this group appears to be the most promising for the establishment of biomarkers at the OSCC, all relevant and respective articles are also discussed in more detail in Table 1 (Tab. 1) and Table 2 (Tab. 2).

iii) The immunological pathway was the subject in a further 56 articles and iv) there were apoptotic mechanisms found in 22 publications (is equivalent to 7% of all relevant articles). 

Figure 5 (Fig. 5) shows the respective distribution of the articles with metabolic and enzymatic accordance.

The articles dealing with the metabolic pathway are summarized in Table 2 (Tab. 2).

## 4 Discussion

### 4.1 Study design and general aspects

Intensive research into the establishment of additive biomarkers in OSCC has now been ongoing for more than 20 years. The first groundbreaking review of biomarkers in OSCC was published in 2003 by Schliephake [22] as a systematic review of 169 articles. However, these 169 articles did not exclusively focus on tumor metabolism. Nevertheless, the conclusion of this review was sobering, as only 12 out of 23 articles dealing with of cell cycle acceleration and proliferation markers showed a significant association with the prognosis of OSCC. Three years later, Lothaire and colleagues provided an updated overview of the critical role of various prognostic factors in OSCC [23]. The author encouraged clinicians and scientists to push ahead with the establishment of additive biomarkers for more precise diagnosis as quickly as possible.

Other reviews on the subject are the articles by Cervino et al. [24], da Silva et al. [25], Dolens et al. [26] and Blatt et al. [27]. In the meantime, various attempts have been made to comprehensively analyze the topic of biomarkers at the OSCC. However, it is not uncommon for the investigations to be rather coarsely focused and each of the investigators had different criteria to filter the articles. Each investigator focused on different aspects of the article search, resulting in a different number of sources to which the investigator referred. For example, it should be mentioned that Cervino et al. [24] summarized 1,884 papers and finally focused on only 8 articles. Dolens et al. [26], on the other hand, included 172 articles and Blatt et al. [27] used 128 studies that met their specific inclusion criteria, among them were proliferative indicators like Ki-67, Cox-2, Cdc7 or ABCB5 [28], [29], [30], [31], [32].

All these excellent review articles are broadly based on the topic of biomarkers at the OSCC. However, the focus of our current survey was on energetic as well as metabolic biomarkers. Therefore, the present investigation would complement these analyses. 

### 4.2 Metabolic aspects, glycolysis in OSCC and its benefit as additive biomarker

Whereas the metabolic-enzymatic pathway for OSCC has already been described quite well by many publications, which is why it should be discussed in more detail in this review. Since Otto Warburg’s groundbreaking experiments on tumor metabolism and the discovery of increased glucose uptake by tumors, many attempts have been made to find valuable additive biomarkers describing this mechanism [9]. Since this major metabolic pathway of glycolysis, which is also significant in OSCC, is very diverse and very complex, so we focused our analysis on metabolic adaptations of gene activity in oral carcinogenesis. Therefore, the metabolic pathway will be the focus of our work in this paper and will be presented in detail in accordance with Figure 1 (Fig. 1).

The metabolic process of glycolysis and associated genes – an early key step in oral carcinogenesis – has been described in many articles. It has been shown that overexpression of different glycolytic enzymes results in a worse prognosis. The most informative and promising markers are GLUT-1 (glucose-transporter type 1), GLUT-3 (glucose-transporter type 3) and HK2 (hexokinase 2), ALDOA (aldolase A, fructose-bisphosphate A), PGK1 (phosphoglycerate kinase 1) or PGAM1 (phosphoglycerate mutase 1). Some representative articles and proteins deserve to be discussed in more detail.

The glycolytic pathway starts with the uptake of glucose into the cells, for which transporters are responsible e.g. GLUTs. GLUTs are part of a carrier family consisting of 13 members. Among these members, GLUT-1 and GLUT-3 are relevant in OSCC. Consequently, GLUTs has been studied quite frequently and it’s not surprising that GLUTs in general are among the most well-studied groups of biomarkers in OSCC [33]. Both GLUT-1 and GLUT-3 [34] can be considered as significant markers of poor prognosis in OSCC. An important meta-analysis regarding GLUT-1 summarized 13 studies with 1,301 subjects published by Li et al. [33]. 

The authors found that an increased expression of GLUT-1 is associated with higher tumor grade (P=0.031), tumor size (P<0.001), and lymph node metastasis (P<0.001) as well as with shorter overall survival in OSCC [33].

The article highlights GLUT-1 as a key enzyme in OSCC prognosis and aggressiveness.

Moreover, some other studies confirm this association in distinguishing between premalignant types and invasive cancer [35]. Beyond that, this general statement about the association between GLUT-1 transporter and OSCC has been reported in several articles [36], [37]. Although GLUT-1 is the main enzyme for the intake of glucose into the cells, there are other important enzymes involved in this pathway downstream of GLUTs. A study regarding ALDOA and PGK1 by [38] shows that a high expression of ALDOA and PGK1 is associated with poor prognosis in OSCC patients and that they can be used as potential markers for predict prognosis and hypoxia in OSCC patients. Both markers are important enzymes in glycolysis. HK2 [39], as well as PGAM1 [40] were found to be relevant OSCC-associated biomarkers. HK2 catalyzes the initial step in the conversion of glucose to glucose-6-phosphate (see Figure 1 (Fig. 1)) [41]. 

Most analyses focused on the development of the primary tumor. Metastasis itself represents a complex cascade in tumor progression that should be characterized in detail in a further review article but is essential for the discussion. The pathway of metastasis must be strictly distinguished from our work. Blatt et al. focused on glycolysis in metastasis in their article [27], which indicates metabolism in OSCC progression/metastasis.

Our analysis supports the results of Blatt et al., but we focus on the metabolism of glycolysis and the enzymes involved in glucose metabolism in primary tumors.

### 4.3 Tumor microenvironment and pH stability

A second metabolic-related and tumor-specific characteristic is the tumor microenvironment. This required factors that affect the pH value e.g. carbonic anhydrase. One crucial enzyme is carbonic anhydrase, a membrane-spanning tumor-associated cell surface glycoprotein that is induced by hypoxia is involved in adaptation to acidosis and in cancer progression [42]. This is because glycolysis itself is able to decrease the extracellular pH value caused by the release of the product of tumor-specific glucose metabolism, namely lactate. This must be fixed by enzymes, produced be the tumor cells.

Intriguingly, all described tumor-related mechanisms required a key regulator protein. The best-investigated one is the hypoxia-induced factor 1 with its alpha subunit (HIF-1α) [43]. HIF itself provides a short half-time of only a few minutes. Therefore, there is an urgent need to establish surrogate markers that have the capacity to reliably describe the content of HIF activation [44]. In this context, the next promising protein that is associated with hypoxia and tumor micromilieu is the prolyl 4-hydroxylase subunit α1 (P4HA1), which encodes the active catalytic component of prolyl 4-hydroxylase (P4H). It is noteworthy to state, this is initially considered as a key enzyme for collagen processing and therefore for the structure of extracellular matrix [38]. On closer inspection, however P4HA1 is involved in HIF stabilization and has earned the term surrogate marker for tumor hypoxia. 

The high metabolic glucose turnover into lactate causes further risks even for the tumor cell. Intracellular acidification is an essential issue, which is an obstacle to tumor DNA replication. At this point, further metabolic proteins are required that contribute to intracellular pH stabilization. The key protein carbonic anhydrase 9 (CAIX), a membrane-spanning tumor-associated cell surface glycoprotein that is induced by hypoxia is involved in adaptation to acidosis and in cancer progression [45]. The interesting GLUT1-CAIX axis has been described in several articles in PubMed^®^ related to OSCC [46], [47], [48].

In addition to glucose, malignant tumors also require other substances to sustain their complex energy utilization. One of these is glutamine, which is needed both as a source of nitrogen to support tumor biomass and additionally for energy production [49], as a consequence, tumor-related increase in glutaminolysis should be discussed and further studies must be analyzed (one study, for example, is that of [50]).

### 4.4 Other metabolic proteins involved in oral carcinogenesis

Remembering the complex network and difficult structure of oral carcinogenesis there is also many peptides that can make metabolic-enzymatic predictions about tumor expression. This very interesting area that is also being investigated and highlighted as follows. In addition to energy supply at the cellular level, the development of a tumor’s own vascular system also plays a decisive role. Whether they intervene in the vascular area such as ET-1, HIF-1 or VEGF, integrated in collagen metabolism like P4HA1, hexoses phosphorylate (hexokinase 2) or cell-surface associated (CAIX), require separate investigations and are only briefly explained and listed here. For example, endothelin (ET-1), a glycolytic enzyme-independent peptide that is involved in microvascular imaging of several organs.

It plays a central role in oral carcinogenesis due to its regulatory axis via miRNA 4893p and TWIST [51]. This phenomenon, together with the fact of increased energy expenditure, leads to the emergence of the epithelial-mesenchymal junction (EMT), another cornerstone of carcinogenesis. On the other hand, the HIF-1 regulated expression of vascular endothelial growth factor A (VEGFA) is a well-known and intensively described process in early oral carcinogenesis. 

The next critical hallmark of OSCC is the development of lymphatic metastases. Reliable biomarkers are the vascular endothelial growth factors C and (VEGF-C/-D). In multivariate analysis, overexpression of VEGF-C and VEGF-D correlated with increased lymphatic vessel density (LVD) and increased lymph node metastasis in OSCC [52].

In addition, biomarkers that can be detected in saliva are also playing an increasingly important role It's about studying biomarkers and another promising approach is to establish salivary biomarkers, as described by [53] for example. This study identified salivary metabolomic biomarkers to predict the prognosis of OSCC based on comprehensive metabolomic analyses. Quantified metabolomics data from unstimulated saliva samples from patients with OSCC were randomly divided into the training and validation groups in Ishikawa et al.’s study [53]. The training data was used to develop a Cox proportional hazards regression model to identify significant metabolites as prognostic factors for overall survival and disease-free survival. Additionally, the validation set was used to develop another Cox proportional hazards regression model using the previously identified metabolites. There were no significant differences between groups in participant characteristics. The concentrations of 5-hydroxylysine (p=0.009) and 3-methylhistidine (p=0.012) were identified as significant prognostic factors for overall survival in the training group. Among them, the concentration of 3-methylhistidine was a significant prognostic factor for overall survival in the validation group (p=0.048). Ishikawa et al.’s results showed that salivary 3-methylhistidine is a prognostic factor for OS in patients with OSCC.

### 4.5 Crosstalk metabolism in immune oncology

Hu et al. underline the key role of both glycolytic relevant enzymes Phosphofructokinase-fructose bisphosphatases PFKFB3 and PFKFB4 with different effects on the prognosis of oral cancer patients with different clinicopathological outcomes [54]. Moreover, PFKFB4 expression was significantly higher in the tumour tissues and may be helpful to discriminate tumours from normal and/or premalignant tissue. The research group among Zhang analysed the role of Nicotinamide N-methyltransferase (NNMT) as a metabolic enzyme catalysing the methylation of nicotinamide (NAM) to generate 1-methyl nicotinamide (MNAM). In addition, NNMT promoted OSCC tumour cell proliferation and migration in vitro [55]. In addition, NNMT was involved in OSCC tumour cell proliferation and migration in vitro and may be considered as critical regulator of EMT in OSCC as well as a prognostic biomarker in OSCC. 

Another interesting aspect is the pivotal function of Histone deacetylase proteins (HDACs) – a family of enzymes that remove acetyl functional groups from histone proteins on DNA. One of them, HDAC6, is located in the cytoplasm and involved in many biological and pathological processes. These are cell migration, the DNA damage response and carcinogenesis, by regulating its substrates. As a consequence, patients, whose tumour intensively expressed HDAC6 showed a 3.248-fold increase mortality risk compared with the low HDAC6 expression group (P=0.003, [56]). Interestingly, levels of HDAC6 may be also a useful prognostic biomarker and offer a novel immune cell-related therapeutic strategy of targeting IL-13 in OSCC. Some investigations into the enzymatic role of carcinogenesis in OSCC is more complex. Song et al. demonstrated the influence of IGF2BP3, encoding for the insulin-like growth factor 2 mRNA-binding protein 3. Their results indicated that OSCC specimen with greater expression levels of IGF2BP3 exhibited significantly shorter overall survival compared to those with lower expression levels (p=0.029). Thus, IGF2BP3 is one possible additional gene/protein in facilitating tumour development and metastasis in vivo [57].

The discovery of the enzyme GGPS1 (geranylgeranyl diphosphate synthase 1) that has so far received little attention is also highly interesting. GGPS1 is a member of the prenyltransferase family and acts as an enzyme for the posttranslational modification of proteins [58], has key roles in signalling pathways like cytoskeletal regulation and intracellular transport and can serve as a biomarker in Hepatocellular carcinoma. Related to OSCC, the authors found significantly higher expressions of GGPS1 in tumour tissues compared to normal oral tissues [59]. Moreover, it is interesting to note that the GGPS1 expressions were very closely negatively linked to overall survival (OS) and disease-free survival (DSS) of the patients of OSCC. Of course, this moves away from classical tumour metabolism but shows the diversity of adaptation mechanisms in oral cancer. 

A similar approach is pursued by Krishna and coworkers. They analysed the prognostic role of Beta 2-Adrenergic Receptor (β2-AR) in OSCC. They have key functions in cAMP-pathway [60]. In Cox proportional hazards model, β2-AR was identified as a prognostic biomarker of OSCC patients. 

A trend can be observed over the entire observation period: while classical biomarkers for energy production and metabolism were still favoured in the first period, other proteins/enzymes that play a supportive role in the complex metabolism of energy production have now followed in recent months. In addition, the cross-linkage to immunologically biologically relevant key proteins has become apparent. This crystallizes that the current research initiatives take into account two relevant Nobel Prizes – on the one hand the one on HIF-1 system and on the other hand the one on immune checkpoint blockade.

## 5 Conclusion

To sum up, our results show that the current approaches in biomarker research in the OSCC are satisfactory compared to 20 years ago. However, if one compares all the pathways we have classified, it turns out that the cell cycle pathway, and here in particular the micro-RNA and the intermediate filaments, have been best studied. Overall, the long-noncoding RNA, the mitosis-associated pathway, the cyclin-dependent kinase, and tumor suppressors have been less described.

Most of the articles also show the fundamental role that glycolysis plays in OSCC prognosis and support Otto Warburg’s pioneering idea in the early 20^th^ century. In our opinion, the specific use of biomarkers in OSCC for an individualized diagnosis and prognosis calculation can be considered as a new milestone in cancer treatment in great accordance with Schilsky’s excellent review [61]. Altogether, considering the multiple research initiatives in the establishment of metabolic and enzymatic biomarkers in OSCC, some key enzymes are crystallizing for use in addition to TNM and grading. These are GLUT-1, CAIX and HK2 – all enzymes that play an essential role in glucose metabolism. As key enzymes, they all excellently characterize the glucose metabolism and thus the energetic situation of the tumor cell: GLUT-1 as the gateway into the cell, HK2 as the initiator of tumor-related metabolism and CAIX for pH stability as well as and for elimination of toxic waste products. The latter can also be described as the gatekeeper of epithelial-mesenchymal transition. An upregulation of these proteins characterizes an increased metabolism and a more aggressive tumor subtype; possible therapeutic options would be targeted strategies against the expression of, for example, the surface enzyme GLUT-1. From a clinical transnational perspective, it can nevertheless be formulated that, at the present time, the most promising additive (metabolic/enzymatic) biomarkers in the form of GLUT-1, HK2 and CAIX can complement the classic TNM system and the grading sufficiency and should support the clinical setting. 

## Notes

### Funding

The authors hereby confirm that they did not receive any financial support in carrying out the research. This research did not receive specific funding from any public, commercial, or for-profit sectors.

The authors acknowledge the financial support of the Open Access Publication Fund of the Martin Luther University Halle-Wittenberg.

### Competing interests

The authors declare that they have no competing interests.

## Figures and Tables

**Table 1:**
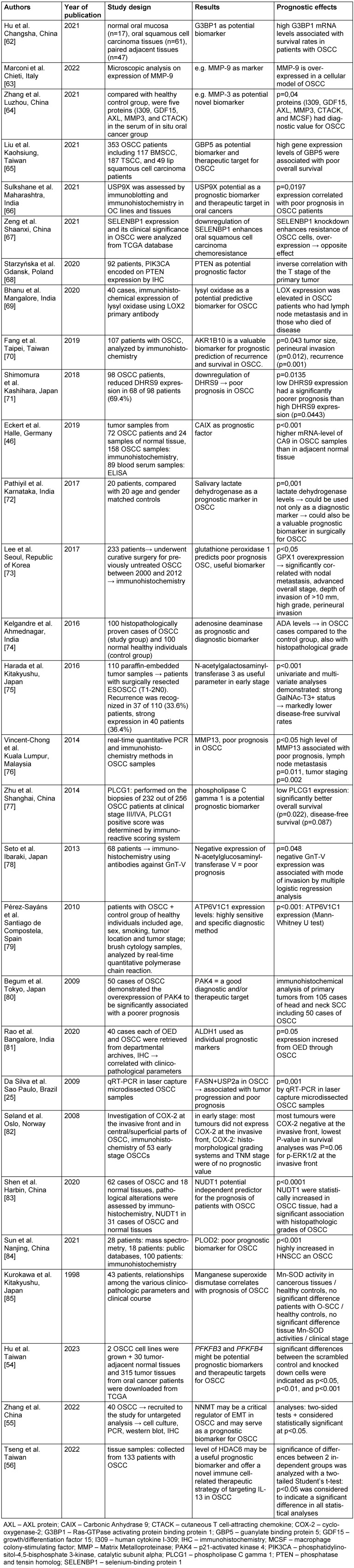
Relevant papers dealing with enzymatic biomarkers in OSCC

**Table 2:**
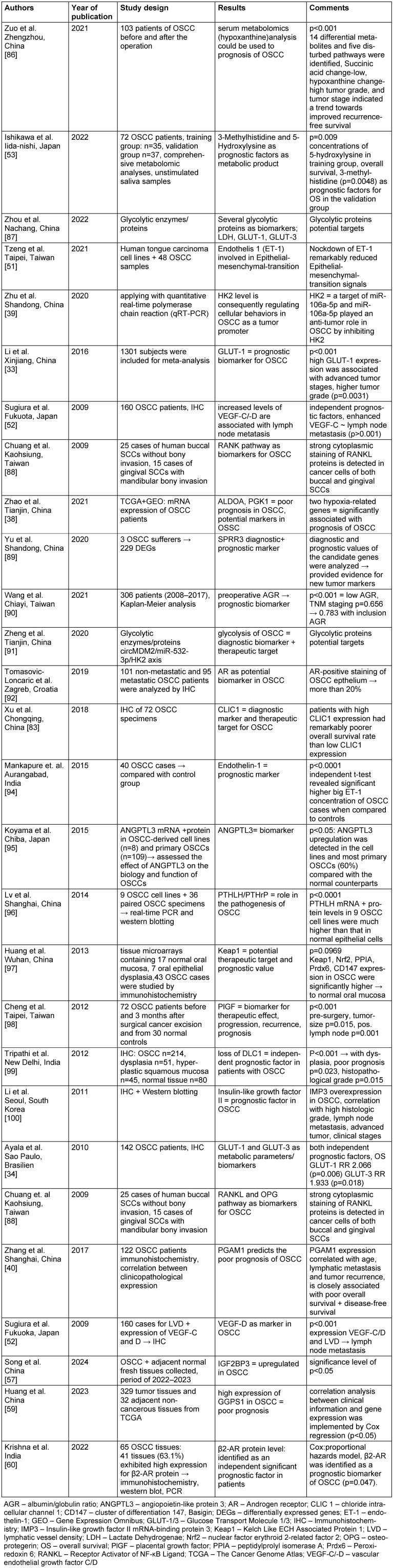
Overview to relevant investigations with respect to metabolic pathways in OSCC
